# Supplementary material for: Identification of Two Novel Circular RNAs Deriving from BCL2L12 and Investigation of Their Potential Value as a Molecular Signature in Colorectal Cancer
Source: Int J Mol Sci. 2020 Nov 23;21(22):8867. doi: 10.3390/ijms21228867 (PMC7709015; doi:10.3390/ijms21228867)

Clip. 1 BQ 20 WL 10 Sequence: circ-1_Ex5extF-Ex3R_F

Clipped length:	188	Samples:	15317	Quality:	0 - 9	
Left clip:	9	Bases:	196		10 - 19	
Right clip:	196	Average spacing:	79.0		20 - 29	
Avg. qual. in clip.:	49.67	Average quality >=	10: 4, 20: 8, 30: 170		>= 30	

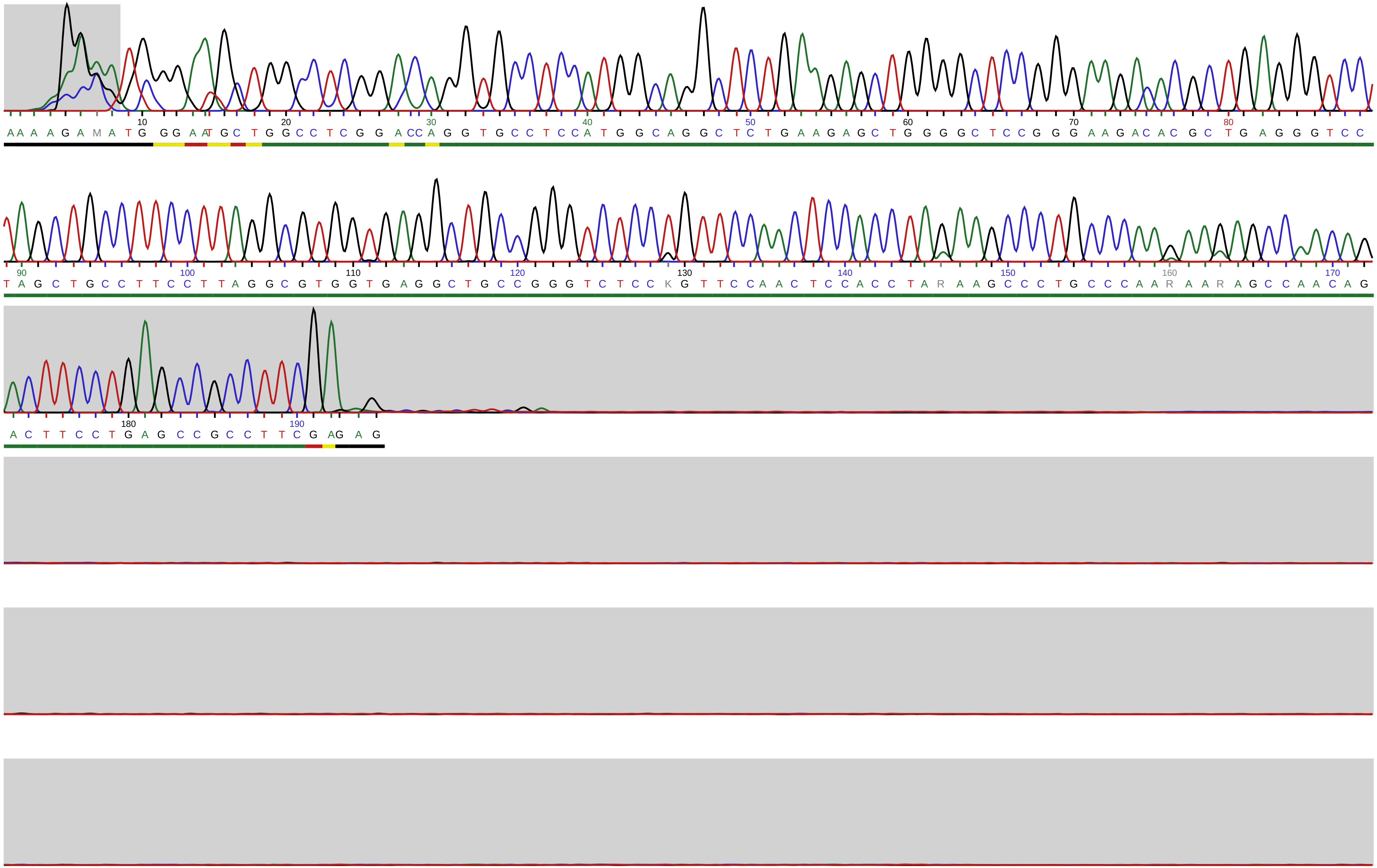

Clip. 1 BQ 20 WL 10 Sequence: circ-1_Ex5extF-Ex3R_R

Clipped length:	188	Samples:	26896	Quality:	0 - 9	
Left clip:	6	Bases:	193		10 - 19	
Right clip:	193	Average spacing:	140.0		20 - 29	
Avg. qual. in clip.:	49.37	Average quality >=	10: 5, 20: 15, 30: 164		>= 30	

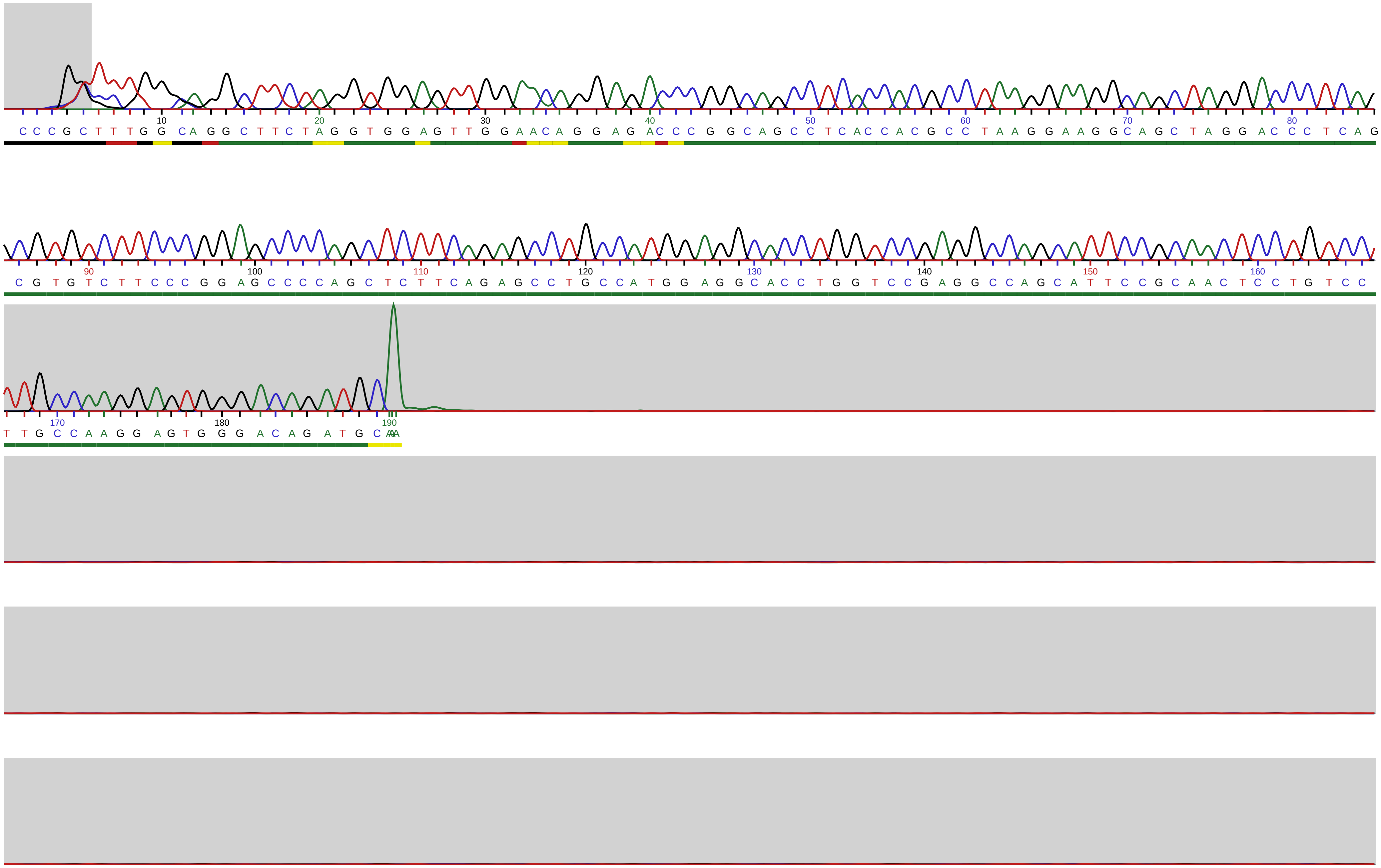

Clip. 1 BQ 20 WL 10 Sequence: circ-1_Ex2F-Ex5R_F

Clipped length:	199	Samples:	29406	Quality:	0 - 9	
Left clip:	120	Bases:	318		10 - 19	
Right clip:	318	Average spacing:	93.0		20 - 29	
Avg. qual. in clip.:	45.3	Average quality >=	10: 60, 20: 55, 30: 168		>= 30	

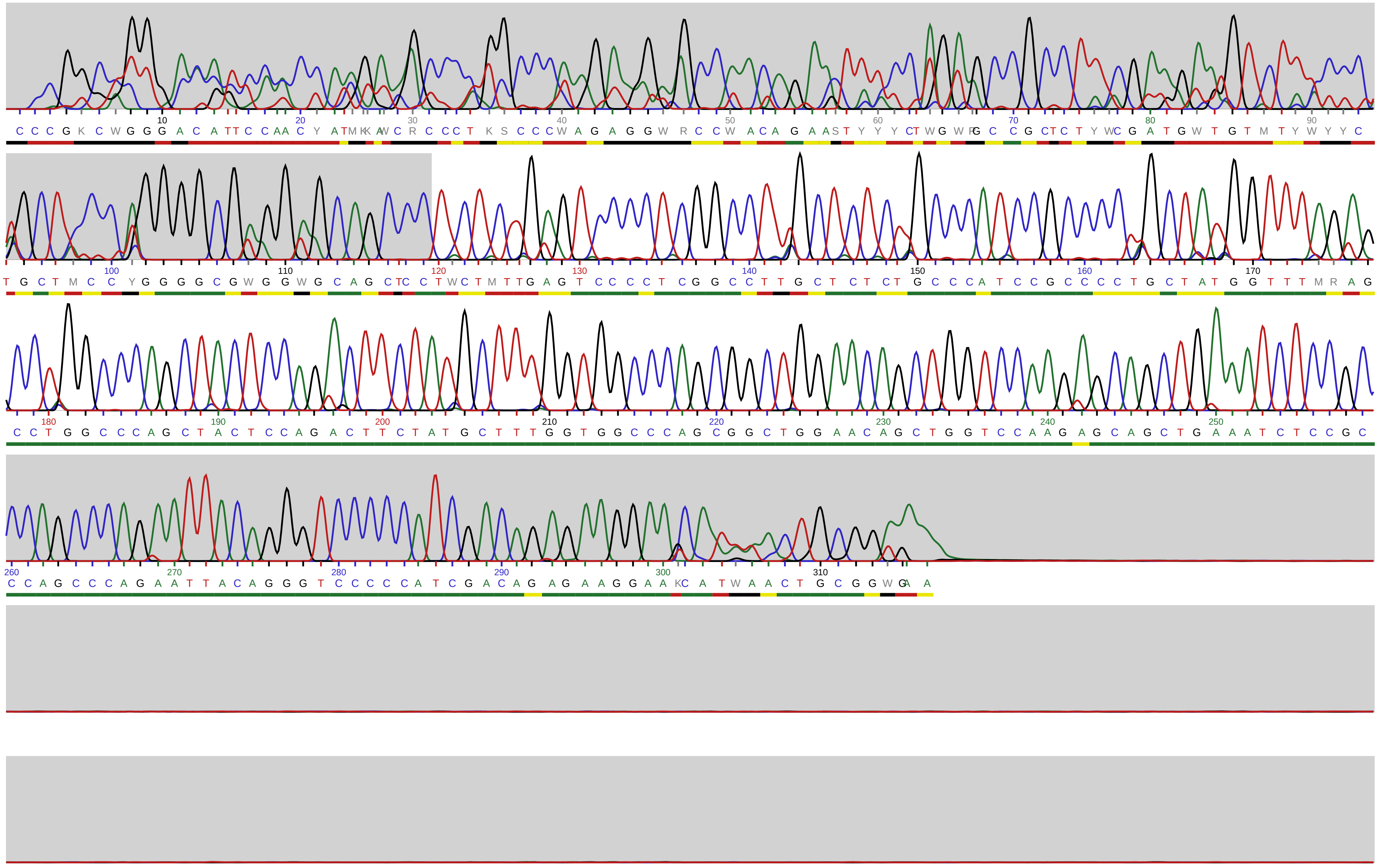

Clip. 1 BQ 20 WL 10 Sequence: circ-1_Ex2F-Ex5R_R

Clipped length:	253	Samples:	25614	Quality:	0 - 9	
Left clip:	56	Bases:	308		10 - 19	
Right clip:	308	Average spacing:	84.0		20 - 29	
Avg. qual. in clip.:	45.91	Average quality >=	10: 29, 20: 62, 30: 202		>= 30	

10.09.2020

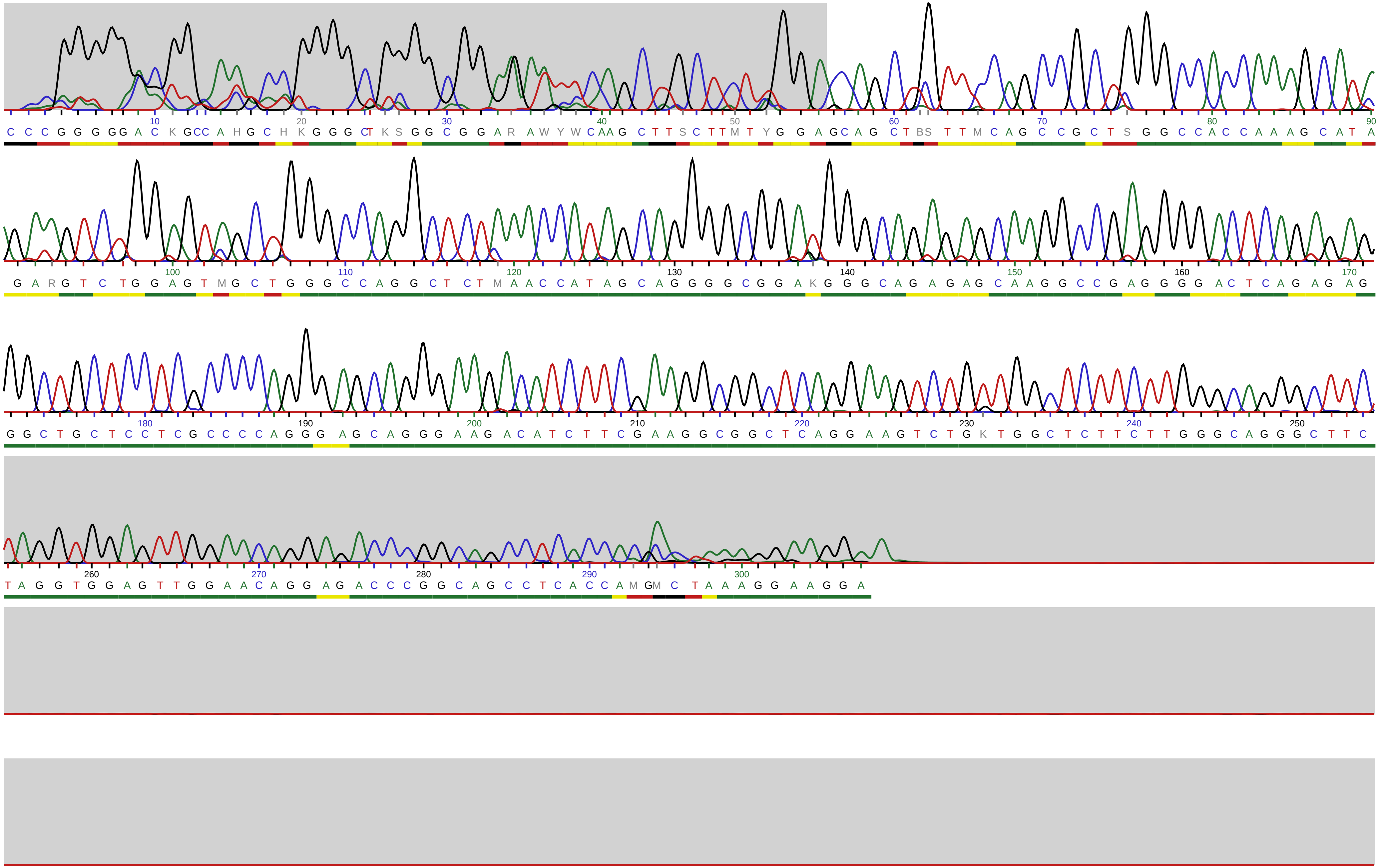

Clip. 1 BQ 20 WL 10 Sequence: circ-2_Ex5F-Ex4R_F

Clipped length:	25	Samples:	12963
Left clip:	76	Bases:	505
Right clip:	100	Average spacing:	26.0
Avg. qual. in clip.:	20.68	Average quality >=	10: 361, 20: 63, 30: 3

Quality: 0 - 9, 10 - 19, 20 - 29, >= 30

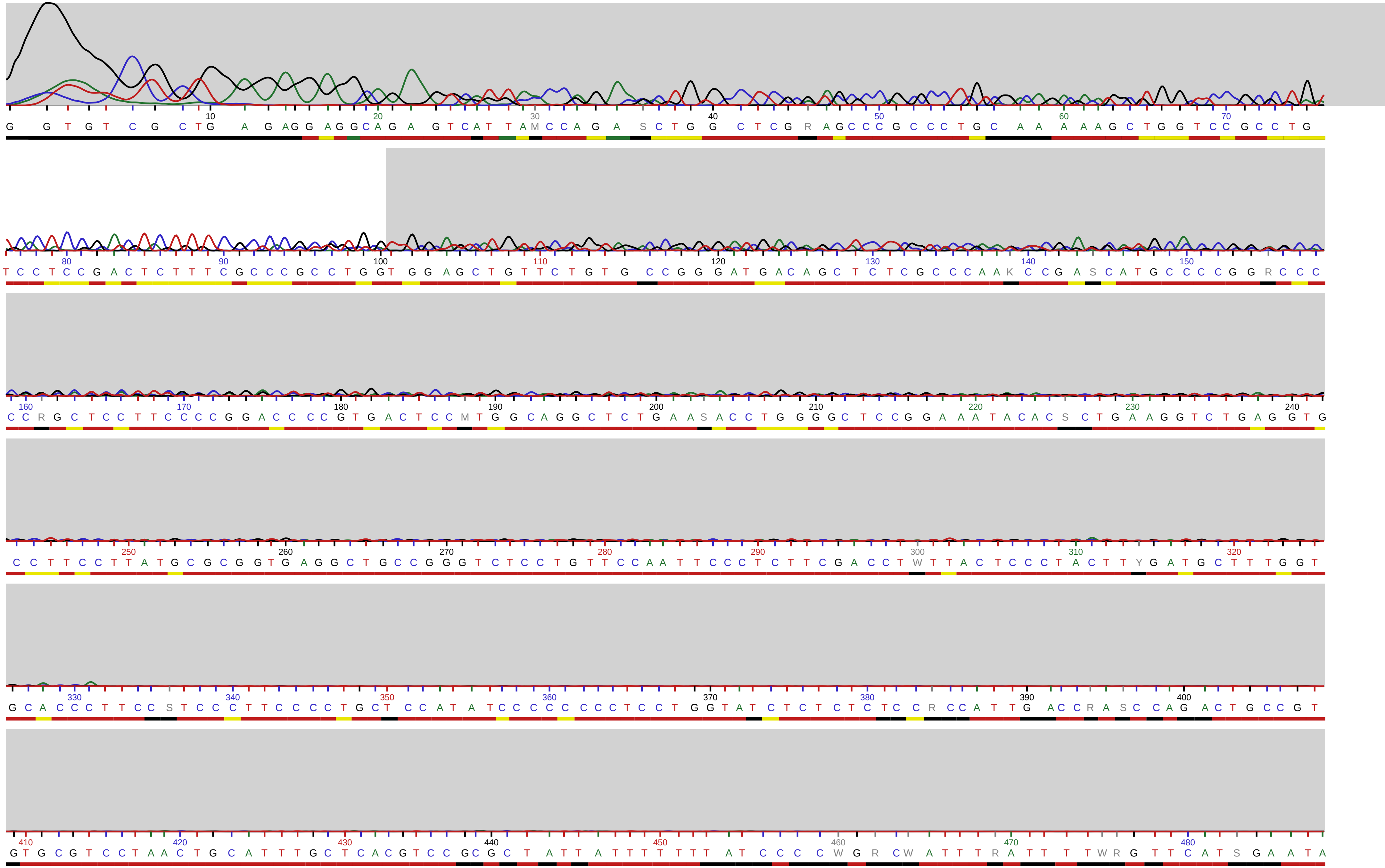

Clip. 1 BQ 20 WL 10 Sequence: circ-2_Ex5F-Ex4R_R

Clipped length:	17	Samples:	12962	Quality:	0 - 9	
Left clip:	119	Bases:	511		10 - 19	
Right clip:	135	Average spacing:	26.0		20 - 29	
Avg. qual. in clip.:	19.64	Average quality >=	10: 335, 20: 46, 30: 1		>= 30	

10.08.2020

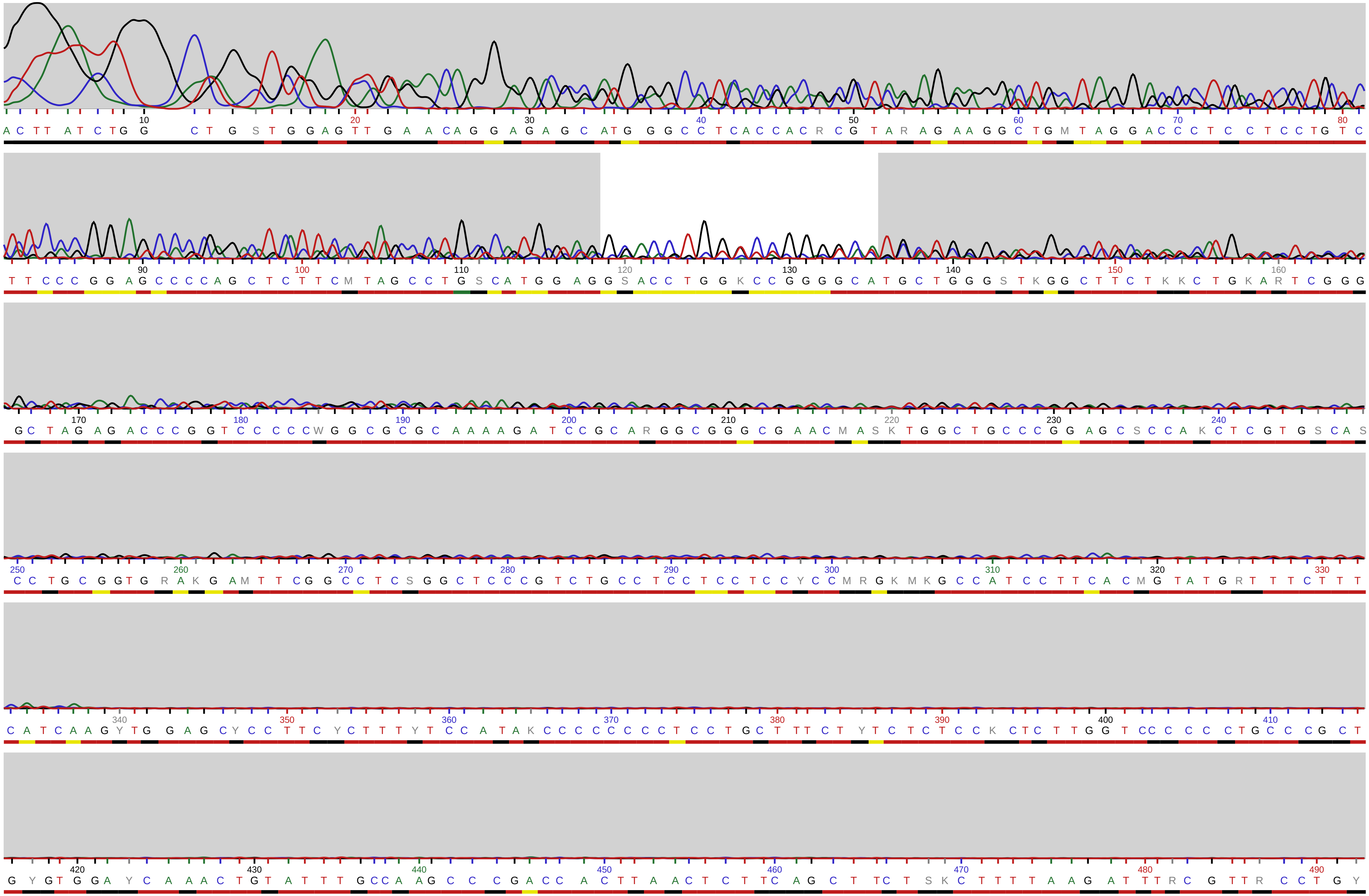

Clip. 1 BQ 20 WL 10 Sequence: circ-2_Ex2F-Ex5R_F

Clipped length:	149	Samples:	29809	Quality:	0 - 9	
Left clip:	19	Bases:	711		10 - 19	
Right clip:	167	Average spacing:	42.0		20 - 29	
Avg. qual. in clip.:	46.71	Average quality >=	10: 272, 20: 152, 30: 130		>= 30	

15.09.2020

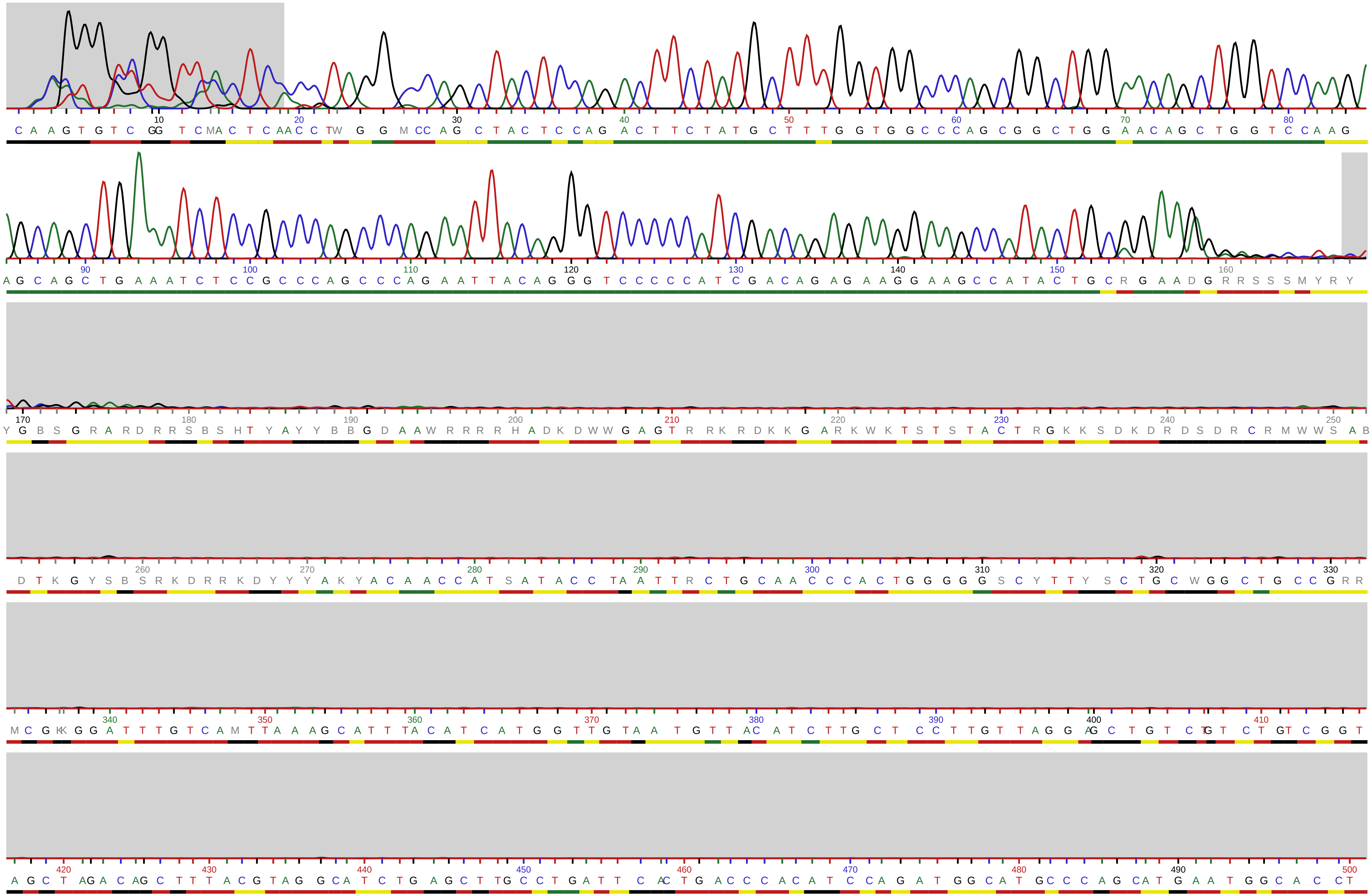

Clip. 1 BQ 20 WL 10 Sequence: circ-2_Ex2F-Ex5R_R

Clipped length:	114	Samples:	15949	Quality: 0 - 9	
Left clip:	52	Bases:	292	10 - 19	
Right clip:	165	Average spacing:	55.0	20 - 29	
Avg. qual. in clip.:	31.28	Average quality >=	10: 109, 20: 64, 30: 56	>= 30	

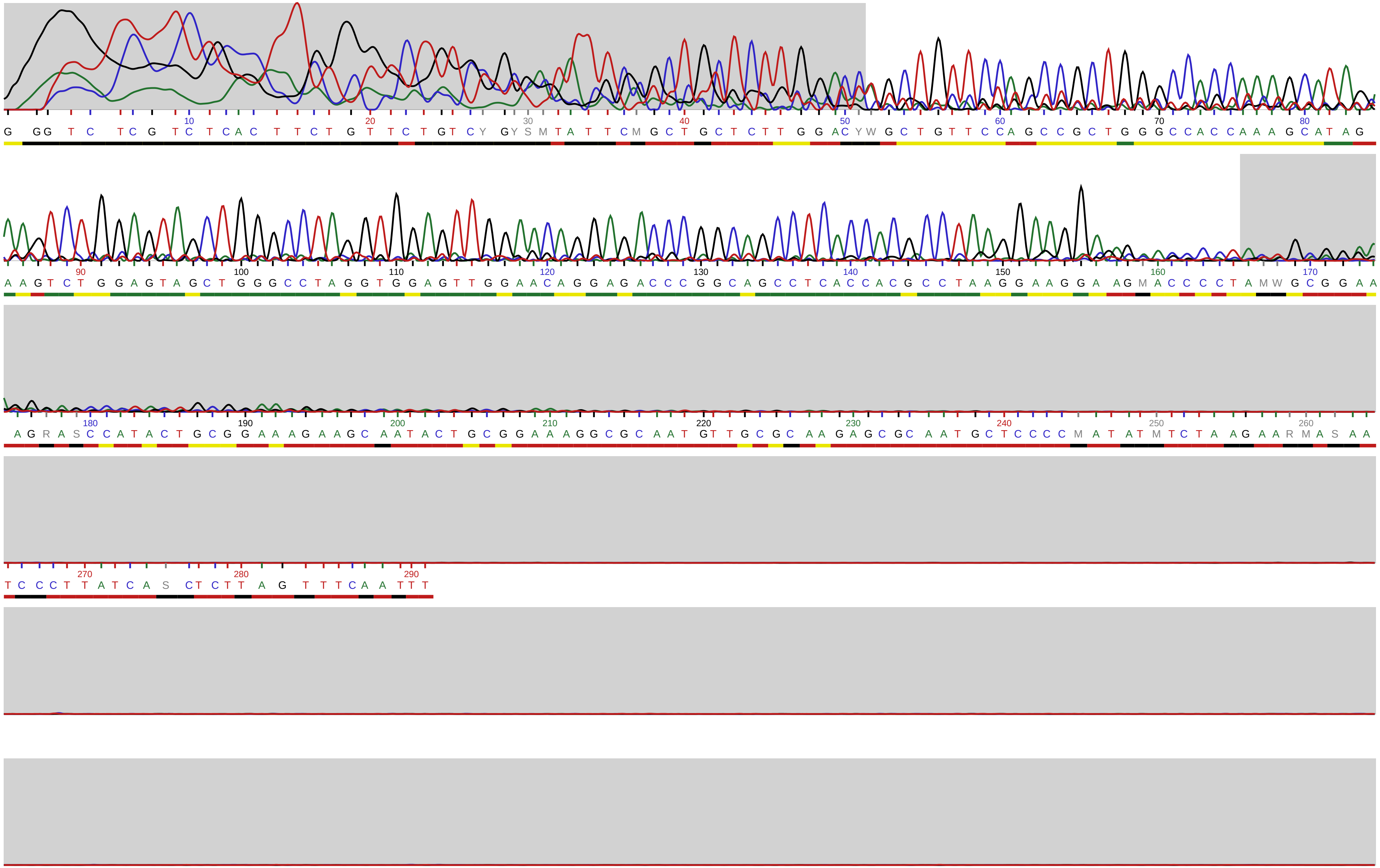

Supplement: Supplementary file 1 [file ijms-21-08867-s001.zip › Supplementary Materials, Data Set 1.pdf]
