# Supplementary material for: Identification of Two Novel Circular RNAs Deriving from BCL2L12 and Investigation of Their Potential Value as a Molecular Signature in Colorectal Cancer
Source: Int J Mol Sci. 2020 Nov 23;21(22):8867. doi: 10.3390/ijms21228867 (PMC7709015; doi:10.3390/ijms21228867)
Supplement: Supplementary file 1 [file ijms-21-08867-s001.zip › Supplementary Tables/Table S1.docx]

**Table S1.** miR-1915-5p–targeted mRNAs that encode proteins implicated in CRC, as predicted using bioinformatical tools.

| **miR-1915-5p–targeted mRNAs** | **Prediction scores** | | | |
| --- | --- | --- | --- | --- |
|  | **TarBase (v.8)** | **miRDB** | **TargetRank** | **miRWalk** |
| ELL-associated factor 1 (*EAF1)* |  | 90 | 62 |  |
| Death-associated protein kinase 1 (*DAPK1)* |  | 86 | 59 |  |
| Histone deacetylase 2 (*HDAC2)* |  | 80 | 60 |  |
| Peroxiredoxin 3 (*PRDX3)* |  | 73 | 60 | 80 |
| Insulin-like growth factor 2 mRNA-binding protein 1 (*IGF2BP1)* | 72 |  | 92 |  |
