# Supplementary material for: Identification of Two Novel Circular RNAs Deriving from BCL2L12 and Investigation of Their Potential Value as a Molecular Signature in Colorectal Cancer
Source: Int J Mol Sci. 2020 Nov 23;21(22):8867. doi: 10.3390/ijms21228867 (PMC7709015; doi:10.3390/ijms21228867)
Supplement: Supplementary file 1 [file ijms-21-08867-s001.zip › Supplementary Tables/Table S2.docx]

**Table S2.** The mutational status^1^ of key genes, microsatellite instability (MSI), and CpG island methylator phenotype (CIMP) of the CRC cell lines used in the current study.

| **Cell lines** | **Genes** | | | | **MSI status** | **CIMP status** |
| --- | --- | --- | --- | --- | --- | --- |
|  | ***TP53*** | ***KRAS*** | ***BRAF*** | ***PIK3CA*** |  |  |
| Caco-2 | p.E204X | wt | wt | wt | MSS | CIMP- |
| COLO 205 | p.Y107fs; p.Y103fs | wt | p.V600E | wt | MSS | CIMP+ |
| DLD-1 | p.S241F | p.G13D | wt | p.E545K; p.D549N | MSI | CIMP+ |
| HCT 116 | wt | p.G13D | wt | p.H1047R | MSI | CIMP+ |
| HT-29 | p.R273H | wt | p.V600E; p.T119Sc | wt | MSS | CIMP+ |
| RKO | wt | wt | p.V600E | p.H1047R | MSI | CIMP+ |
| SW 620 | p.R273H; p.P309S | p.G12V | wt | wt | MSS | CIMP- |

^1^ Mutated or wild-type (wt).
