# Supplementary material for: Identification of Two Novel Circular RNAs Deriving from BCL2L12 and Investigation of Their Potential Value as a Molecular Signature in Colorectal Cancer
Source: Int J Mol Sci. 2020 Nov 23;21(22):8867. doi: 10.3390/ijms21228867 (PMC7709015; doi:10.3390/ijms21228867)
Supplement: Supplementary file 1 [file ijms-21-08867-s001.zip › Supplementary Tables/Table S3.docx]

**Table S3.** First-round PCR and semi-nested PCR primers, used for the identification and/or Sanger sequencing of *BCL2L12* circRNAs.

| **Direction** | **Name** | **Sequence (5′→3′)** | **Length (nt**^1^**)** | **T_m_ (^o^C)** |
| --- | --- | --- | --- | --- |
| **Forward** | Ex5 ext F | GCATCTGTCCCACTCCTTGG | 20 | 62 |
|  | Ex2F | CCTTCCTTAGGCGTGGTGAG | 20 | 61 |
|  | Ex5F | GAAGGAAGCCATACTGCGGA | 20 | 61 |
| **Reverse** | Ex3R | CGAAGGCGGCTCAGGAA | 17 | 62 |
|  | Ex4R | GGGCCACCAAAGCATAGAAG | 20 | 60 |
|  | Ex5R | TCCGCAGTATGGCTTCCTTC | 20 | 61 |

^1^ Nucleotides.
