# Supplementary material for: Identification of Two Novel Circular RNAs Deriving from BCL2L12 and Investigation of Their Potential Value as a Molecular Signature in Colorectal Cancer
Source: Int J Mol Sci. 2020 Nov 23;21(22):8867. doi: 10.3390/ijms21228867 (PMC7709015; doi:10.3390/ijms21228867)
Supplement: Supplementary file 1 [file ijms-21-08867-s001.zip › Supplementary Tables/Table S4.docx]

**Table S4.** First-round PCR and semi-nested PCR primer pairs, used for the identification of *BCL2L12* circRNAs.

| **circRNA** | **Primer name** | | **Amplicon size (bp**^1^**)** |
| --- | --- | --- | --- |
|  | **Forward** | **Reverse** |  |
| circ-BCL2L12-1 | Ex5 ext F | Ex5R | 447 |
|  | Ex2F | Ex5R | 327 |
|  | Ex5 ext F | Ex3R | 217 |
| circ-BCL2L12-2 | Ex5F | Ex5R | 462 |
|  | Ex2F | Ex5R | 184 |
|  | Ex5F | Ex4R | 366 |

^1^ Base pairs.
