# Supplementary material for: Identification of Two Novel Circular RNAs Deriving from BCL2L12 and Investigation of Their Potential Value as a Molecular Signature in Colorectal Cancer
Source: Int J Mol Sci. 2020 Nov 23;21(22):8867. doi: 10.3390/ijms21228867 (PMC7709015; doi:10.3390/ijms21228867)
Supplement: Supplementary file 1 [file ijms-21-08867-s001.zip › Supplementary Tables/Table S5.docx]

**Table S5.** Primers used in PCR-based pre-amplification and real-time qPCR, for the quantification of *BCL2L12* circRNAs.

|  | **Direction** | **Name** | **Sequence (5′→3′)** | **Length (nt**^1^**)** | **T_m_ (^o^C)** |
| --- | --- | --- | --- | --- | --- |
| **Pre-amplification** | **Forward** | HPRT1 F | AACCTCTCGGCTTTCCCG | 18 | 61 |
|  |  | 6/2F | GCCTCGGACCAGGTGC | 16 | 64 |
|  |  | 5/6F | TTAACCAGAAGCTGGCCTCG | 20 | 61 |
|  | **Reverse** | HPRT1 R | CAGTGCTTTGATGTAATCCAGCAG | 24 | 59 |
|  |  | 3inR | CTGTTGGCTCTTCTTGGGCA | 20 | 61 |
|  |  | 5/4R | GACCCTGTAATTCTGGGCTGG | 21 | 61 |
| **Real-time PCR** | **Forward** | HPRT1 2F | TGGAAAGGGTGTTTATTCCTCAT | 23 | 57 |
|  |  | 2F | GGGTCCTAGCTGCCTTCCTT | 20 | 63 |
|  |  | 2/4F | CAACTCCACCTAGGCCCAG | 19 | 62 |
|  | **Reverse** | HPRT1 3R | ATGTAATCCAGCAGGTCAGCAA | 22 | 60 |
|  |  | 3R | CTCTTCTTGGGCAGGGCTT | 19 | 61 |
|  |  | 4R | GACCAGCTGTTCCAGCCG | 18 | 63 |

^1^ Nucleotides.
