# Supplementary material for: Identification of Two Novel Circular RNAs Deriving from BCL2L12 and Investigation of Their Potential Value as a Molecular Signature in Colorectal Cancer
Source: Int J Mol Sci. 2020 Nov 23;21(22):8867. doi: 10.3390/ijms21228867 (PMC7709015; doi:10.3390/ijms21228867)
Supplement: Supplementary file 1 [file ijms-21-08867-s001.zip › Supplementary Tables/Table S6.docx]

**Table S6.** Pre-amplification and real-time qPCR primer pairs, used for the quantification of *BCL2L12* circRNAs.

| **Gene** | **Transcript** | **Primer name** | | **Amplicon size (bp**^1^**)** |
| --- | --- | --- | --- | --- |
|  |  | **Forward** | **Reverse** |  |
| *HPRT1* | (unique) | HPRT1 F | HPRT1 R | 370 |
|  | (unique) | HPRT1 2F | HPRT1 3R | 151 |
| *BCL2L12* | circ_BCL2L12_1 | 6/2F | 3inR | 153 |
|  |  | 2F | 3R | 83 |
|  | circ_BCL2L12_2 | 5/6F | 5/4R | 373 |
|  |  | 2/4F | 4R | 69 |

^1^ Base pairs.
