# Supplementary figures and images for: Identification of Two Novel Circular RNAs Deriving from BCL2L12 and Investigation of Their Potential Value as a Molecular Signature in Colorectal Cancer
Source: Int J Mol Sci. 2020 Nov 23;21(22):8867. doi: 10.3390/ijms21228867 (PMC7709015; doi:10.3390/ijms21228867)

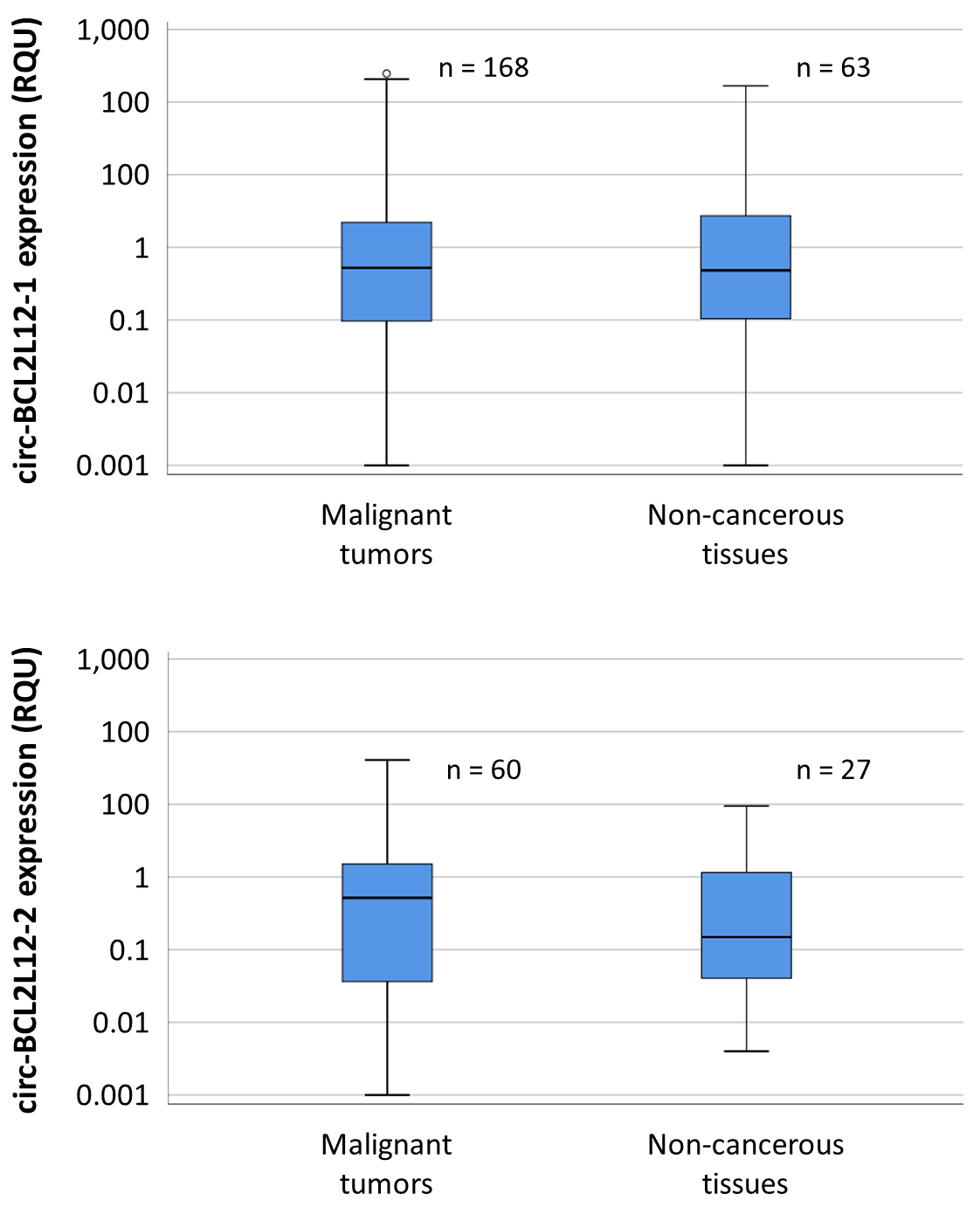

Supplement: Supplementary file 1 [file ijms-21-08867-s001.zip › Supplementary Figures/Figure S1.tif]

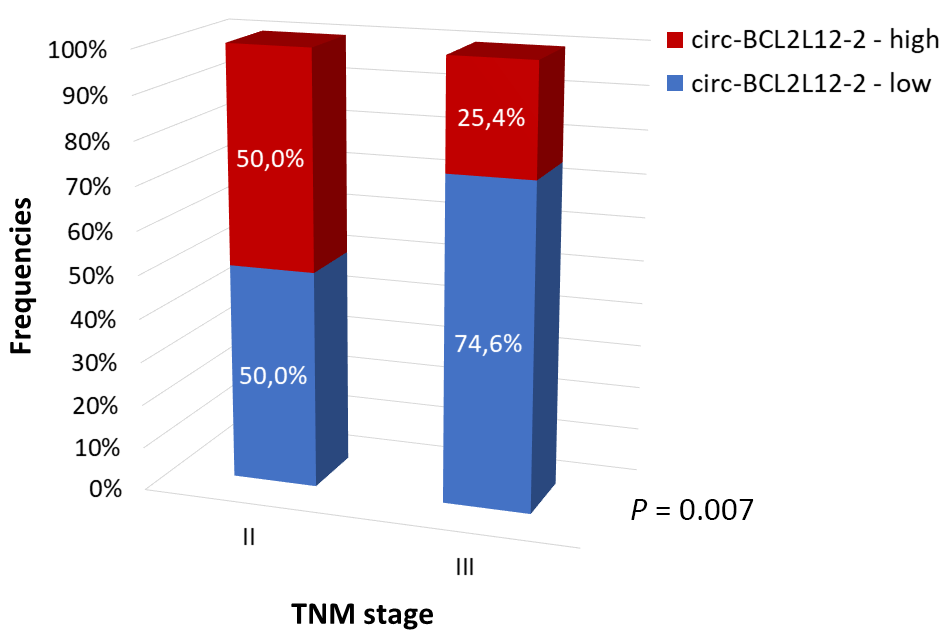

Supplement: Supplementary file 1 [file ijms-21-08867-s001.zip › Supplementary Figures/Figure S2.tif]
